# Outcome reporting bias in randomized-controlled trials investigating antipsychotic drugs

**DOI:** 10.1038/tp.2017.203

**Published:** 2017-09-12

**Authors:** M Lancee, C M C Lemmens, R S Kahn, C H Vinkers, J J Luykx

**Affiliations:** 1 Department of Psychiatry, Brain Center Rudolf Magnus, University Medical Center Utrecht, Utrecht University, Utrecht, The Netherlands; 2 Department of Psychiatry, Icahn School of Medicine, Mount Sinai, NY, USA; 3 Department of Translational Neuroscience, Brain Center Rudolf Magnus, University Medical Center Utrecht, Utrecht University, Utrecht, The Netherlands; 4 Department of Psychiatry, ZNA Hospitals, Antwerp, Belgium

## Abstract

Recent literature hints that outcomes of clinical trials in medicine are selectively reported. If applicable to psychotic disorders, such bias would jeopardize the reliability of randomized clinical trials (RCTs) investigating antipsychotics and thus their extrapolation to clinical practice. We therefore comprehensively examined outcome reporting bias in RCTs of antipsychotic drugs by a systematic review of prespecified outcomes on ClinicalTrials.gov records of RCTs investigating antipsychotic drugs in schizophrenia and schizoaffective disorder between 1 January 2006 and 31 December 2013. These outcomes were compared with outcomes published in scientific journals. Our primary outcome measure was concordance between prespecified and published outcomes; secondary outcome measures included outcome modifications on ClinicalTrials.gov after trial inception and the effects of funding source and directionality of results on record adherence. Of the 48 RCTs, 85% did not fully adhere to the prespecified outcomes. Discrepancies between prespecified and published outcomes were found in 23% of RCTs for primary outcomes, whereas 81% of RCTs had at least one secondary outcome non-reported, newly introduced, or changed to a primary outcome in the respective publication. In total, 14% of primary and 44% of secondary prespecified outcomes were modified after trial initiation. Neither funding source (*P*=0.60) nor directionality of the RCT results (*P*=0.10) impacted ClinicalTrials.gov record adherence. Finally, the number of published safety endpoints (*N*=335) exceeded the number of prespecified safety outcomes by 5.5 fold. We conclude that RCTs investigating antipsychotic drugs suffer from substantial outcome reporting bias and offer suggestions to both monitor and limit such bias in the future.

## Introduction

Even though clinical trials constitute the backbone of evidence-based medicine, a substantial proportion of their outcomes have proven overstated, flawed or difficult to reproduce.^1, 2, 3^ To ensure the transparency and reliability of clinical trial research, the National Institutes of Health (NIH) and the Food and Drug Administration (FDA) created a national clinical trials registry and results database (ClinicalTrials.gov) in 2000.^4^ This registry now constitutes the largest online clinical trials database.^5^ Submission of study results to ClinicalTrials.gov is mandatory for US-based clinical trials following the 2007 FDA Amendments act,^6^ which aims to prevent outcome reporting bias, defined as selective publication of clinical trial outcomes.^7, 8^

While the above-mentioned regulations have been in place for about a decade, evidence hints that selective outcome reporting still occurs in several fields of medicine, including oncology, cardiology, rheumatology and gastroenterology.^9, 10, 11, 12, 13, 14, 15^ For example, results for at least one outcome mentioned in Methods section remain unreported in ~30% of clinical trials.^16^ Selective publication of clinical trial results has substantial negative impact as it renders the interpretation of scientific findings less reliable. Consequently, meta-analyses risk producing biased or inflated results and thereby introduce bias in the translation of published evidence into clinical practice.^8, 17^

In the field of psychiatry, landmark studies using data from the FDA reported an excess of positive results in randomized-controlled trials (RCTs) investigating antidepressants.^7, 18^ Using a similar approach, Turner *et al.* found evidence of outcome reporting bias in 20 published antipsychotic trials, when comparing FDA reviews of antipsychotics with published trial data.^19^ Additional studies investigating selective publication of outcomes in RCTs of antipsychotic agents, however, are lacking, a striking knowledge gap given the widespread and ever increasing use of these agents globally.^20, 21, 22, 23^ For example, no study had systematically compared outcomes of trial methods prespecified on ClinicalTrials.gov to outcomes reported in the respective scientific publications.

Antipsychotics are prescribed to ~1 and 1.5% of the UK and US populations, respectively.^24, 25^ Assessing the degree to which outcome reporting bias plays a role in RCTs investigating antipsychotics may help gauge the soundness of their results and guide future studies in minimizing risks of selective reporting.^26^ We therefore aimed to elucidate the nature and degree of outcome reporting bias in RCTs of antipsychotics. To that end, we systematically compared outcome measures of such RCTs prespecified on ClinicalTrials.gov with their scientific publications. Moreover, we examined whether any updating of trial outcomes occurred during or after the trial period had been carried out and whether funding source and directionality of RCT results are associated with poor adherence to ClinicalTrials.gov records.

## Materials and methods

### Search strategy and selection criteria

To systematically retrieve and analyze applicable studies we applied the PRISMA guidelines.^27^ Thus, we first systematically searched for clinical trial registration on ClinicalTrials.gov (referred to as ‘record’ or ‘prespecified’ data) on 18 July 2015, using the search terms '(schizophrenia OR schizoaffective disorder) AND antipsychotic'. Results were limited to phase II–IV RCTs investigating antipsychotics, for which study enrollment was no longer possible (‘closed’ status on ClinicalTrials.gov).

All RCTs had to be received by ClinicalTrials.gov between 1 January 2006 and 31 December 2013. The starting date was chosen in light of the mandated WHO registration of RCTs since 2006.^28^ Titles and abstracts of retrieved records were screened. We did not constrain our search by RCT design (for example, open label studies were allowed). Exclusion criteria were: indications other than schizophrenia or schizoaffective disorder (for example, bipolar disorder), antipsychotics not listed as main treatment, use of non-FDA approved antipsychotics, use of one or more concomitant interventional drug(s) besides antipsychotics (for example, antidepressants), ongoing and prematurely terminated RCTs, and non-randomized RCTs. Matching publications were retrieved by searching PubMed with ClinicalTrials.gov Identifiers (NCT numbers) on 17 November 2015. Data in the publications are referred to as ‘published’ or ‘reported’. The end date was selected since median time period to publication after completion of trials is nearly two years.^29^ Titles and abstracts of retrieved publications were compared with information in ClinicalTrials.gov records to verify whether they matched. To avoid overlap between study populations in our systematic review, one publication per trial record was selected. If multiple publications on PubMed could be linked to a single NCT number, we included the publication in which the methods corresponded most accurately with the trial record on ClinicalTrials.gov. For records lacking matching publications on PubMed, EMBASE and CINAHL were screened to ensure the inclusion of all published RCTs in our study. If no publication was found in either of the databases, the RCT was excluded.

### Data extraction

Baseline characteristics were extracted from ClinicalTrials.gov for all RCTs, including sample size, date of first record, funding source and trial start and completion dates. All data were retrieved and evaluated by two independent authors (ML and CMCL). Discordant judgments were resolved in consensus meetings between both authors.

Primary and secondary outcomes as stated in trial records were reviewed and compared with the accompanying publications by two authors (ML and CMCL). Discrepancies were resolved by discussions with the other co-authors. Primary outcomes were defined as outcomes stated as such in either ClinicalTrials.gov records or publications. Outcomes described in either of these as ‘other outcome measures’ were considered secondary outcomes.

On ClinicalTrials.gov, the most recent versions of prespecified trial outcomes were extracted per RCT. Deleted and added primary and secondary outcomes on ClinicalTrials.gov between the first and last updated versions in the database were extracted. Primary and secondary outcomes in the publications were sorted into the following categories after comparing these between ClinicalTrials.gov and PubMed: published and unpublished outcomes that were prespecified in the trial record; published outcomes that had not been prespecified on ClinicalTrials.gov; and outcomes that had changed status from primary to secondary or vice versa in the publication relative to the record. To allow for conservative estimations of outcome reporting bias, slightly different phrasing of similar outcome variables between records and publications was allowed (for example, metabolic syndrome clinical findings were considered equal to BMI and weight circumference, see Supplementary Table 1). In addition, results and *P*-values of primary outcomes were extracted. Furthermore, published results of primary outcomes were rated negative (in accordance with the null hypothesis) or positive. With regard to reporting of safety and tolerability outcomes, substantial variation was present between records, ranging from few unspecified to several detailed outcomes. Therefore, safety and tolerability outcomes defined as such by authors of the publications (including (serious) adverse events, vital signs and laboratory parameters) were separately reviewed from other secondary outcomes.

To examine the timing of altered prespecified outcomes on ClinicalTrials.gov, we compared the dates of the first received records and subsequent changes after trial inception, termination and/or publication.

### Statistical analysis

We used SPSS software (version 23.0) for our statistical analyses (IBM, Armonk, NY, USA, 2015). We computed the following descriptive statistics for continuous study characteristics: means, medians and s.d. To further disentangle the degree to which outcome reporting bias applies to secondary outcomes, Spearman’s correlation was used to calculate the correlation between the number of secondary outcomes prespecified on ClinicalTrials.gov and the number of non-reported secondary outcomes in the publications. To examine whether the directionality of efficacy outcomes (positive or negative) influences authors’ decisions to publish outcome measures, one-sided Fisher exact test was used, comparing the proportions of newly added positive primary outcomes with the proportions of positive results in the overall group of primary outcomes. The same statistical test was used to compare record adherence in industry-funded with non-industry-funded RCTs. Significance threshold (alpha) for these statistical tests was set at 0.05.

## Results

### Baseline characteristics

**Figure 1:**
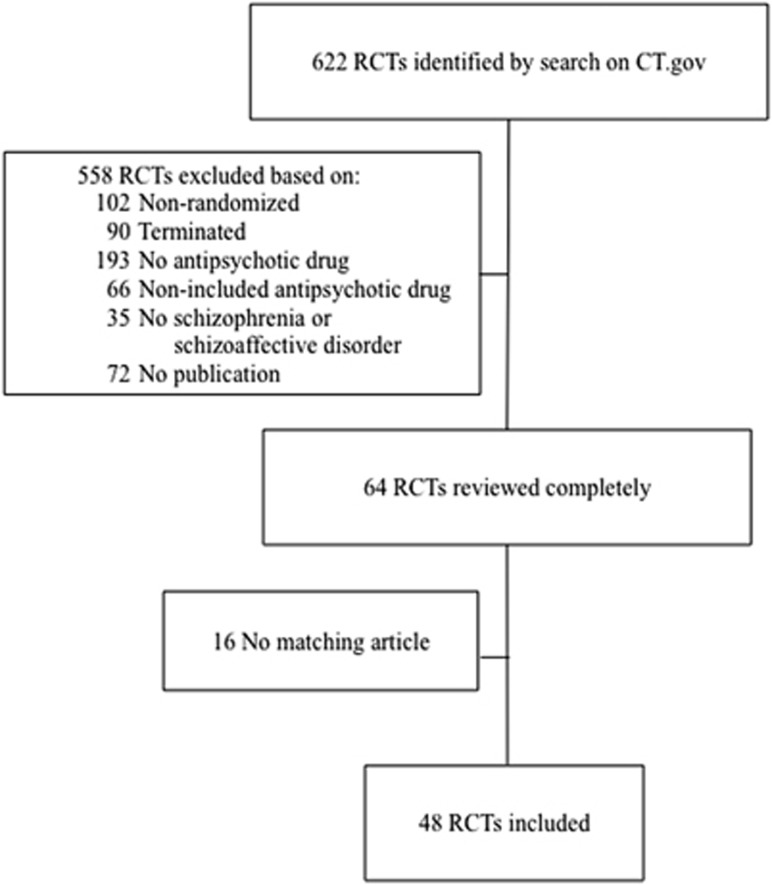
Flowchart illustrating the process of RCT selection.

A total of 622 RCTs were retrieved, 558 of which were excluded because they did not meet inclusion criteria (Figure 1). Of the completed RCTs that examined antipsychotics in schizophrenia or schizoaffective disorder (*N*=136), no publication on PubMed was found for 72 ClinicalTrials.gov records. An additional 16 studies missed a record-matching publication on PubMed. Consequently, 48 RCTs were included in this study (Figure 1 and Supplementary Table 2). The median number of participants of these 48 RCTs was 319.5 (range: 58–18 154). A total of 13 antipsychotic agents were investigated in the included RCTs, the most frequently investigated antipsychotic being paliperidone (23%) (Table 1 and Supplementary Table 2). The majority of RCTs were phase III (63%), industry-funded (81%) and multicenter (73%).

### Adherence to study record

In total, 7 of the 48 included RCTs (15%) provided published outcomes entirely in line with the primary and secondary outcomes prespecified on ClinicalTrials.gov. Of the industry-sponsored RCTs (*N*=39), six (15%) fully adhered to their records, which was similar to non-industry-funded RCTs (11% *P*=0.61).

**Figure 2:**
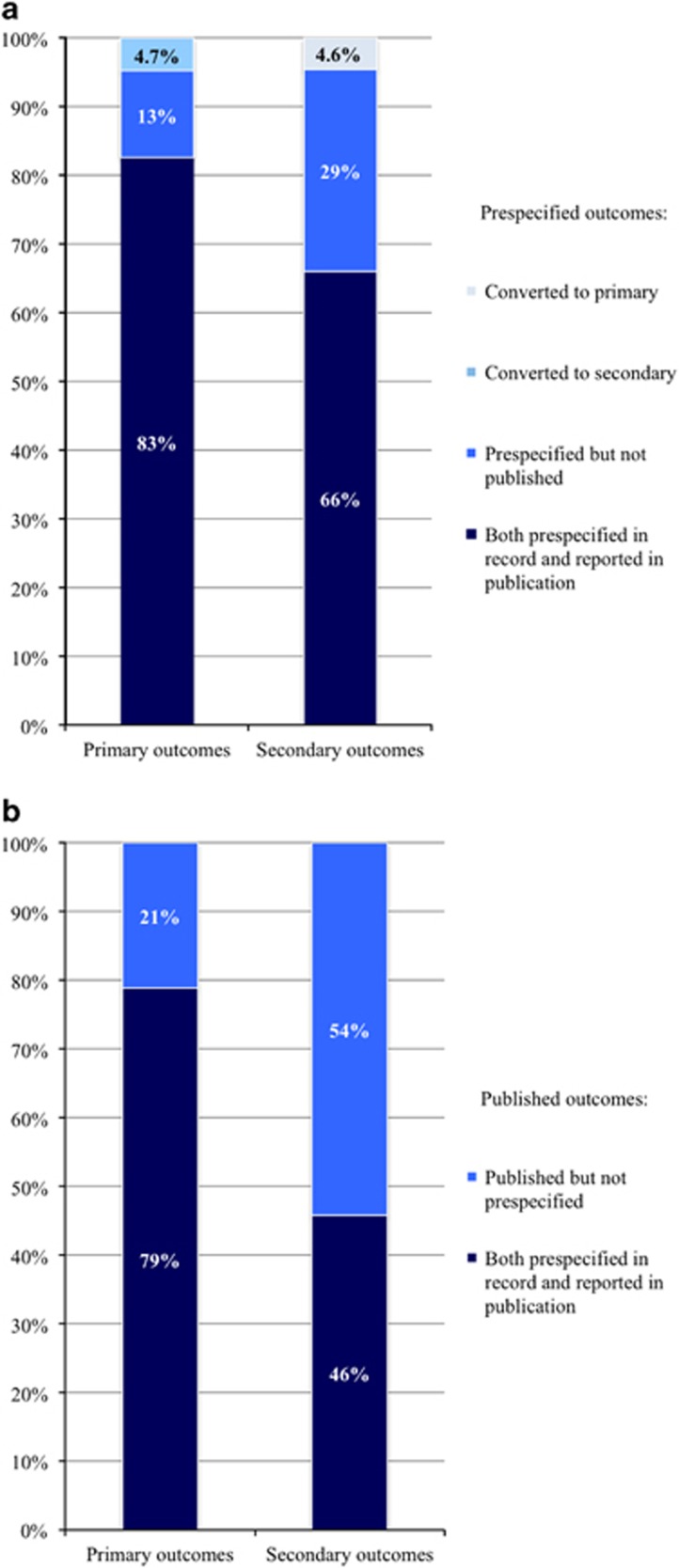
Consistencies between primary and secondary outcomes in records and publications. (**a**) Converted or non-reported outcomes in publications relative to records. (**b**) Reported outcomes not prespecified in records relative to publications. Data are presented as 100% stacked columns. The left bar in (**a**) denotes that of 63 primary outcomes, three (4.7%) were converted to secondary outcomes in the publications relative to the ClinicalTrials.gov records; eight (13%) were found in ClinicalTrials.gov records but not in the respective publications; and 52 (83%) were both prespecified in records and reported in publications. The right bar denotes that out of 197 secondary outcomes, nine (4.6%) were converted to primary outcomes; 58 (29%) were prespecified on ClinicalTrials.gov but not reported in the respective publications; and 130 (66%) were both prespecified in the ClinicalTrials.gov records and reported in the publications. The left bar in (**b**) denotes that out of 66 primary outcomes, 14 (21%) were reported in the publications but had not been prespecified in their ClinicalTrials.gov records; and 52 (79%) were both prespecified in records and reported in publications. The right bar denotes that of 284 published secondary outcomes 154 (54%) had not been prespecified in the ClinicalTrials.gov records and 130 (46%) were both prespecified in records and published in publications.

**Figure 3:**
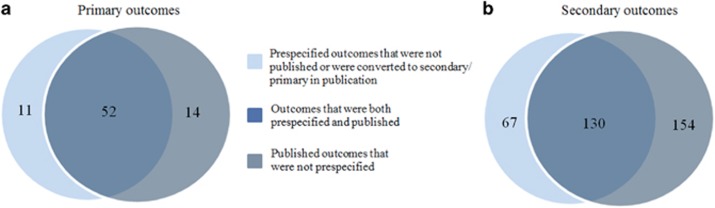
Venn diagram of consistencies and discrepancies in outcome reporting between ClinicalTrials.gov records and publications. (**a**) Primary outcomes. (**b**) Secondary outcomes. Figure illustrating the extent of overlap in primary (**a**) and secondary outcomes (**b**) between ClinicalTrials.gov records and study publications (dark blue). Discrepancies included prespecified outcomes not published or converted to secondary in publication (light blue) and published outcomes that had not been prespecified in ClinicalTrials.gov records (grey).

A total of 66 primary outcomes were published in the 48 RCTs, with nine RCTs introducing multiple primary outcomes. For the majority of RCTs, the primary outcome(s) concerned efficacy (83%); the remaining primary outcomes concerned safety endpoints. In 11 RCTs (23%) discrepancies were found between the prespecified and published primary outcomes, with a total of 25 altered primary outcomes (Figures 2 and 3, Supplementary Table 2). These changes included prespecified primary outcomes that were not mentioned in the publication (*N*=8, Table 2); publication of primary outcomes that had not been prespecified (*N*=14); and publication of prespecified primary outcomes as secondary outcomes (*N*=3). Again, discrepancy rates for industry-sponsored RCTs (26%) were similar to non-industry-funded RCTs (22% *P*=0.60).

Of the 66 published primary outcomes, 45 primary outcomes (68%) were deemed to be positive findings. Of the newly added primary outcomes (*N*=14), seven outcomes (50%) were positive, which was not significantly different from the percentage of positive results in the prespecified group of primary outcomes (68% *P*=0.10).

A total of 284 secondary outcomes were published with an average of 5.9 (s.d. 4.4) secondary outcomes per RCT. In 39 RCTs (81%), one or more discrepancies were found in secondary outcomes between records and publications (Figures 2 and 3, Supplementary Table 2). Of all 197 prespecified secondary outcomes, 29% were not reported in the applicable publication (Table 2). Conversely, in 37 RCTs (77%), new secondary outcomes were introduced in the publications that had not been specified on ClinicalTrials.gov, the total number of newly introduced secondary outcomes being 154, with an average of 3.2 (s.d. 3.3) per included RCT. Consequently, 54% of all published secondary outcomes had not been prespecified. In four of the 48 studies (8.3%), at least one outcome defined in the record as secondary was converted to a primary outcome in the publication. Finally, we found a significant positive correlation between the number of prespecified secondary outcomes and the number of non-reported secondary outcomes in the accompanying publication (Spearman rho=0.67; *P*=1.47 × 10^−7^), indicating that researchers registering large numbers of secondary outcomes are least likely to fully adhere to their ClincalTrials.gov records.

With regard to safety and tolerability, 53 out of 74 prespecified outcomes (72%) were included in the accompanying publications. A total of 335 new safety and tolerability outcomes were introduced in the publications, exceeding the planned number by >5.5 fold.

### Registering or updating of study record

In total, 23 studies (48%) registered the trial record on ClinicalTrials.gov after trial initiation. Of these, 11 studies were received by ClinicalTrials.gov in the same month as trial inception. For the 25 RCTs registering their records after trial initiation, almost two years elapsed between trial inception and registration on ClinicalTrials.gov (range 1–82 months). Eight RCTs submitted trial records to ClinicalTrials.gov after the trial had been completed.

**Figure 4:**
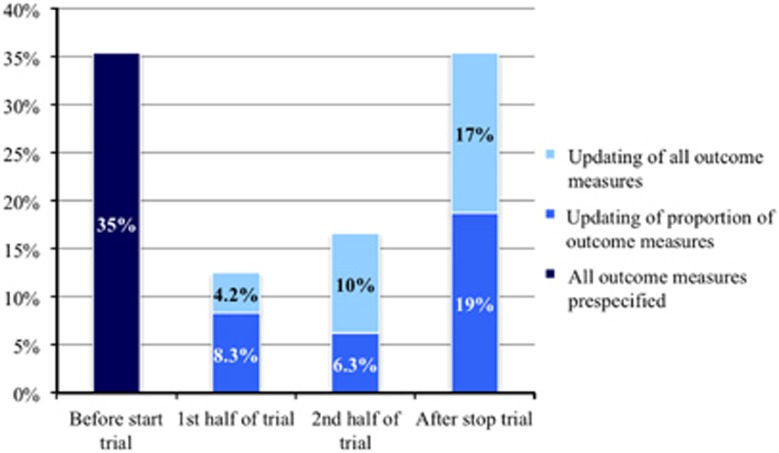
Registration of RCTs relative to trial initiation and updating of outcomes after trial initiation. Figure depicting the extent to which outcome sections on ClinicalTrials.gov are altered after trial initiation. The *x*-axis denotes the trial period during which either a proportion of outcomes are updated or all outcome measures are newly uploaded to the ClinicalTrials.gov records. The *y*-axis denotes percentages relative to all outcomes. Data are presented as stacked columns. Figures in the stacks represent percentages of RCTs. The first bar on the left denotes that of 48 studies 17 (35%) had all outcome measures registered before the trial start date. The second bar denotes that two studies (4.2%) updated all of their outcome measures in the first half of the trial and four studies (8.3%) did this for a proportion of their outcomes. The third bar denotes that five studies (10%) updated all of their outcome measures in the second half of the trial and three studies (6.3%) updated a proportion of their outcomes in the second half of their trial. The fourth bar denotes that eight studies (17%) updated all of their outcomes and nine studies (19%) a proportion of their outcomes after the trial stop date.

In 35% of the RCTs, all final primary and secondary outcomes were registered on ClinicalTrials.gov before the study start date (Figure 4). The remaining studies either updated their registered outcomes during the trial period (29%), or updated/enlisted all their outcomes only after trial initiation (35% Figure 4).

A total of 20 RCTs (42%) modified outcomes in the record after the registered trial initiation date, with on average two modifications (s.d. 3.8) in primary and secondary outcomes per included RCT. Alterations in records included removal of registered outcomes (*N*=22) and added outcomes (*N*=74). Consequently, 14% of registered primary outcomes and 44% of registered secondary outcomes across 48 RCTs were changed after trial initiation. No publication mentioned that registered outcomes had been amended during the trial period. On average, modifications in records were conducted 36 months after the date of record registration (range 3–84 months, s.d. 18).

## Discussion

This study signals discrepancies between outcomes prespecified on ClinicalTrials.gov and published outcomes in 85% of RCTs investigating antipsychotic drugs, with 23% of RCTs showing discrepancies in primary outcomes and 81% in secondary outcomes. Discrepancies included prespecified outcomes not mentioned in the publications and publication of outcomes that had not been prespecified in trial records. Approximately half of RCTs were registered on ClinicalTrials.gov after trial initiation, with frequent modifications in primary and secondary prespecified outcomes, on average around 36 months after trial registration. Approximately 17% of the RCTs were registered on ClinicalTrials.gov after trial completion. We were unable to detect any impact of the directionality of trial efficacy outcomes (that is, whether the results were positive or negative) on the decision by authors to add or omit outcome measures in publications. Funding (industry vs non-industry) did not influence the degree of selective outcome reporting either.

These results indicate that substantial outcome reporting bias is present in RCTs investigating antipsychotics in the treatment of schizophrenia and schizoaffective disorder. Several researcher-related and journal-related explanations may underlie our findings. Our data hint that the mere existence of trial registers, such as ClinicalTrials.gov, does not eradicate selective outcome reporting. This is in line with studies showing that approximately a quarter of medical trials does not pre-register study protocols.^9, 30^ A study assessing the accuracy of trial registration showed that primary outcomes and key secondary outcomes are often missing at the time of registration,^28^ thus limiting the possibility of monitoring outcome reporting bias. In a large 2005 survey of 519 trials, researchers were found not to report certain outcomes based on space limitations or a lack of clinical relevance or statistical significance.^16^ Another cause of selective outcome reporting may be that researchers underestimate the consequences of modifying prespecified outcomes.^31^ Issues such as missing data, delays in data collection and concerns about the validity of trial results render study outcomes more likely to remain unreported.^31^ Trial registration is not mandated by a range of medical journals, while retrospectively registered trials are frequently accepted for publication.^32, 33, 34^ Once adequately registered, however, few journal editors compare submitted protocols with trial manuscripts. Moreover, discrepancies between prespecified and published information do not appear to constitute reasons for rejection by editors.^30, 33^ We suggest that scientific journals mandate researchers to explicitly address discrepancies between planned and reported outcomes in their publications, thus reducing the likelihood of inconsistent reporting between records and scientific publications.^12, 30^

We find no evidence that positive outcomes (that is, those rejecting the null hypothesis) were intentionally added to records after trial inception, as positive results were not significantly overrepresented within added outcomes relative to the percentage of registered positive outcomes. Although this analysis does not fully probe all possible ethical considerations in the process from record to publication, scientific misconduct, or a propensity to selectively publish positive results, does not seem to drive our findings. Similarly, we find no evidence that industry sponsoring influences the likelihood of outcome reporting bias.

To our knowledge, the current study is the first to demonstrate selective outcome reporting in the field of antipsychotics’ trials by comparison of ClinicalTrials.gov data with published outcomes. Our findings are in line with recent studies that compared prespecified with published outcomes in other fields of medicine, where discrepancies in primary outcomes were detected in ~30% of RCTs.^9, 35^ One of the few other studies investigating reporting bias of both primary and secondary outcomes, in oncology, found similar incidences of selective reporting of primary and secondary outcomes.^11^ With regard to the timely registration of trials, our data are in line with a recent study showing that 70% of trials registered their protocols in a public trial registry before starting the trial.^34^

Strengths of our methods include detailed assessment of both primary and secondary outcomes in records and publications, whereas previous investigations similar to ours focused on primary end points. Moreover, we included all RCTs on antipsychotics within the defined timeframe, minimizing the risk of selection bias. Furthermore, we examined temporal patterns in records after trial initiation and retrospective registration of trials to refine the understanding of selective outcome reporting. All data were separately reviewed by two investigators and discrepancies were resolved by discussions with the other co-authors, minimizing the risk of inter-observer variation impacting study findings. Finally, as per our conservative method, we did not consider slightly differently phrased safety outcomes in publications as outcome reporting modifications. Future studies could more strictly interpret such different phrasing as discrepancies between ClinicalTrials.gov records and publications, which could result in even higher detected rates of outcome reporting bias in antipsychotic trials. Nonetheless, a limitation may be that the number of retrieved published RCTs is relatively small, which may have limited statistical power to detect an impact of the directionality of RCT results on selective reporting of outcomes. This may be due to our method not successfully linking one or more ClinicalTrials.gov records to their respective publications.^36^ Alternatively, since the median time to publication after trial completion is estimated at 21 months,^29^ some ClinicalTrials.gov records dating from 2013 or 2012 may have resulted in reports published after 17 November 2015. We examined the number of scientific articles published after 17 November 2015 that could be linked to our ClinicalTrials.gov records by carrying out a search shortly before publication (on 17 May 2017). We thus retrieved seven publications not included in our primary analyses. Given the similar rates of outcome reporting bias in these studies (Supplementary Table 4) compared with the studies included in our primary analyses, inclusion of these additional studies would not have changed our findings. Another limitation may be that no first-generation antipsychotics were included, since little research has been conducted on these agents over recent years. The absence of this medication subgroup in our analyses nonetheless hampers the extrapolation of our conclusions to such agents. Furthermore, we selected only one publication per corresponding trial record, even when multiple publications were linked to a single NCT number. Researchers may have chosen to publish certain outcomes in additional publications, a possibility that cannot be refuted on the basis of the current method. Finally, we did not examine discrepancies between the directionality of the results posted on ClinicalTrials.gov and those in the accompanying scientific publications. Future research should disentangle the extent to which non-significant or negative study results influence decisions to publish trial results. Moreover, FDA data could be used to examine possible correlations between the significance of trial results and outcome reporting bias.

In the current systematic review, we find evidence of substantial outcome reporting bias in RCTs investigating antipsychotic drugs. We recommend that authors of scientific journal articles address inconsistencies between prespecified and reported outcomes in their publications. Journal editors in turn should strictly enforce this in order to enhance the reliability of trial results. Initiatives such as the International Committee of Medical Journal Editors’ trial registration policy to publish study protocols prospectively,^37^ and comprehensive studies into outcome reporting bias will increase awareness amongst researchers. Finally, we highly encourage monitoring of selective outcome reporting, for example, initiatives such as the COMPare Trials Project.^38^

## Figures and Tables

**Table 1:** Baseline characteristics of included RCTs

*Variables*	*No. of trials (*N=*48)*	*%*
*Results posted on ClinicalTrials.gov*		
Yes	27	56
No	21	44
		
*Phase of Study (as designated)*		
II	1	2.8
II/III	1	2.8
III	30	63
IV	16	33
		
*Antipsychotic*		
Paliperidone	11	23
Aripiprazole	8	17
Quetiapine	7	15
Lurisadone	6	13
Risperidone	5	10
Olanzapine	3	6.3
Brexpiprazole	2	4.2
Sertindole	1	2.1
Asenapine	1	2.1
Ziprasidone	1	2.1
Cariprazine	1	2.1
Clozapine	1	2.1
Sertindole/quetiapine	1	2.1
		
*Funding Source*		
Industry	39	81
Other	9	19
		
*Mono/multicenter*		
Multicenter	35	73
Monocenter	7	15
Not specified	6	13

**Table 2:** Discrepancies between prespecified and published primary/secondary outcomes

	*No. of trials with discrepancies (%)*	*Total no. of discrepancies (% of prespecified/published outcomes)*	*Mean no. of discrepancies per included trial (*N=*48)*
*Primary outcomes*			
Prespecified outcome not published	4 (8%)	8/63 (13%)	0.2
Prespecified outcome converted to secondary outcome in publication	3 (6%)	3/63 (5%)	0.1
Published outcomes not prespecified	10 (21%)	14/66 (21%)	0.3

*Secondary outcomes*			
Prespecified outcome not published	18 (38%)	58/197 (29%)	1.2
Prespecified outcome converted to primary outcome in publication	4 (8.3%)	9/197 (5%)	0.2
Published outcomes not prespecified	37 (77%)	154/284 (54%)	3.2
