# Supplementary material for: Outcome reporting bias in randomized-controlled trials investigating antipsychotic drugs
Source: Transl Psychiatry. 2017 Sep 12;7(9):e1232–. doi: 10.1038/tp.2017.203 (PMC5639247; doi:10.1038/tp.2017.203)
Supplement: Supplementary Table 2 [file tp2017203x2.docx]

| **Study No.** | **Study Reference** | **NCT Number** | **Type of antipsychotic** | **Sample size in publication** | **Funding** | **Mono-/ multicenter** | **Continent(s)** | **Discrepancies in primary outcome(s)** | **Discrepancies in secondary outcome(s)** |
| --- | --- | --- | --- | --- | --- | --- | --- | --- | --- |
| 1 | [McEvoy JP](http://www.ncbi.nlm.nih.gov.proxy.library.uu.nl/pubmed/?term=McEvoy%20JP%5BAuthor%5D&cauthor=true&cauthor_uid=23473350), [Citrome L](http://www.ncbi.nlm.nih.gov.proxy.library.uu.nl/pubmed/?term=Citrome%20L%5BAuthor%5D&cauthor=true&cauthor_uid=23473350), [Hernandez D](http://www.ncbi.nlm.nih.gov.proxy.library.uu.nl/pubmed/?term=Hernandez%20D%5BAuthor%5D&cauthor=true&cauthor_uid=23473350) et al. Effectiveness of lurasidone in patients with schizophrenia or schizoaffective disorder switched from other antipsychotics: a randomized, 6-week, open-label study. [J Clin Psychiatry.](http://www.ncbi.nlm.nih.gov.proxy.library.uu.nl/pubmed/23473350) 2013 Feb;74(2):170-9. | 01143077 | Lurasidon | 244 | Industry | Multicenter (27) | N-America | No | Yes |
| 2 | [Citrome L](http://www.ncbi.nlm.nih.gov.proxy.library.uu.nl/pubmed/?term=Citrome%20L%5BAuthor%5D&cauthor=true&cauthor_uid=22395527), [Cucchiaro J](http://www.ncbi.nlm.nih.gov.proxy.library.uu.nl/pubmed/?term=Cucchiaro%20J%5BAuthor%5D&cauthor=true&cauthor_uid=22395527), [Sarma K](http://www.ncbi.nlm.nih.gov.proxy.library.uu.nl/pubmed/?term=Sarma%20K%5BAuthor%5D&cauthor=true&cauthor_uid=22395527) et al. Long-term safety and tolerability of lurasidone in schizophrenia: a 12-month, double-blind, active-controlled study. [Int Clin Psychopharmacol.](http://www.ncbi.nlm.nih.gov.proxy.library.uu.nl/pubmed/?term=NCT00641745) 2012 May;27(3):165-76 | 01190254 | Lurasidon | 629 | Industry | Multicenter (73) | N-America; S-America; Europe; Africa; Asia | No | Yes |
| 3 | [Findling RL](http://www.ncbi.nlm.nih.gov.proxy.library.uu.nl/pubmed/?term=Findling%20RL%5BAuthor%5D&cauthor=true&cauthor_uid=26091193), [Landbloom RP](http://www.ncbi.nlm.nih.gov.proxy.library.uu.nl/pubmed/?term=Landbloom%20RP%5BAuthor%5D&cauthor=true&cauthor_uid=26091193), [Mackle M](http://www.ncbi.nlm.nih.gov.proxy.library.uu.nl/pubmed/?term=Mackle%20M%5BAuthor%5D&cauthor=true&cauthor_uid=26091193) et al. Safety and Efficacy from an 8 Week Double-Blind Trial and a 26 Week Open-Label Extension of Asenapine in Adolescents with Schizophrenia.  [J Child Adolesc Psychopharmacol.](http://www.ncbi.nlm.nih.gov.proxy.library.uu.nl/pubmed/?term=NCT01190254) 2015 Jun;25(5):384-96. | 00797277 | Asenapine | 306 | Industry | Monocenter | Asia | No | Yes |
| 4 | [Huang CL](http://www.ncbi.nlm.nih.gov.proxy.library.uu.nl/pubmed/?term=Huang%20CL%5BAuthor%5D&cauthor=true&cauthor_uid=25791540), [Hwang TJ](http://www.ncbi.nlm.nih.gov.proxy.library.uu.nl/pubmed/?term=Hwang%20TJ%5BAuthor%5D&cauthor=true&cauthor_uid=25791540), [Chen YH](http://www.ncbi.nlm.nih.gov.proxy.library.uu.nl/pubmed/?term=Chen%20YH%5BAuthor%5D&cauthor=true&cauthor_uid=25791540) et al. Intramuscular olanzapine versus intramuscular haloperidol plus lorazepam for the treatment of acute schizophrenia with agitation: An open-label, randomized controlled trial. [J Formos Med Assoc.](http://www.ncbi.nlm.nih.gov.proxy.library.uu.nl/pubmed/?term=NCT00797277) 2015 May;114(5):438-45. | 00797277 | Olanzapine | 67 | Other | Monocenter | Asia | No | Yes |
| 5 | [McEvoy JP](http://www.ncbi.nlm.nih.gov.proxy.library.uu.nl/pubmed/?term=McEvoy%20JP%5BAuthor%5D&cauthor=true&cauthor_uid=24846035), [Byerly M](http://www.ncbi.nlm.nih.gov.proxy.library.uu.nl/pubmed/?term=Byerly%20M%5BAuthor%5D&cauthor=true&cauthor_uid=24846035), [Hamer RM](http://www.ncbi.nlm.nih.gov.proxy.library.uu.nl/pubmed/?term=Hamer%20RM%5BAuthor%5D&cauthor=true&cauthor_uid=24846035) et al. Effectiveness of paliperidone palmitate vs haloperidol decanoate for maintenance treatment of schizophrenia: a randomized clinical trial. [JAMA.](http://www.ncbi.nlm.nih.gov.proxy.library.uu.nl/pubmed/?term=NCT01136772) 2014 May 21;311(19):1978-87. | 01136772 | Paliperidon | 311 | Other;  NIH | Multicenter (22) | N-America | No | No |

| 6 | [Pandina G](http://www.ncbi.nlm.nih.gov.proxy.library.uu.nl/pubmed/?term=Pandina%20G%5BAuthor%5D&cauthor=true&cauthor_uid=21092748), [Lane R](http://www.ncbi.nlm.nih.gov.proxy.library.uu.nl/pubmed/?term=Lane%20R%5BAuthor%5D&cauthor=true&cauthor_uid=21092748), [Gopal S](http://www.ncbi.nlm.nih.gov.proxy.library.uu.nl/pubmed/?term=Gopal%20S%5BAuthor%5D&cauthor=true&cauthor_uid=21092748) et al. A double-blind study of paliperidone palmitate and risperidone long-acting injectable in adults with schizophrenia. [Prog Neuropsychopharmacol Biol Psychiatry.](http://www.ncbi.nlm.nih.gov.proxy.library.uu.nl/pubmed/21092748) 2011 Jan 15;35(1):218-26. | 00589914 | Paliperidon | 1220 | Industry | Multicenter (87) | N-America; Europe; Asia | No | Yes |
| --- | --- | --- | --- | --- | --- | --- | --- | --- | --- |
| 7 | [Harvey PD](http://www.ncbi.nlm.nih.gov.proxy.library.uu.nl/pubmed/?term=Harvey%20PD%5BAuthor%5D&cauthor=true&cauthor_uid=24035633), [Siu CO](http://www.ncbi.nlm.nih.gov.proxy.library.uu.nl/pubmed/?term=Siu%20CO%5BAuthor%5D&cauthor=true&cauthor_uid=24035633), [Hsu J](http://www.ncbi.nlm.nih.gov.proxy.library.uu.nl/pubmed/?term=Hsu%20J%5BAuthor%5D&cauthor=true&cauthor_uid=24035633) et al. Effect of lurasidone on neurocognitive performance in patients with schizophrenia: a short-term placebo- and active-controlled study followed by a 6-month double-blind extension. [Eur Neuropsychopharmacol.](http://www.ncbi.nlm.nih.gov.proxy.library.uu.nl/pubmed/24035633) 2013 Nov;23(11):1373-82. | 00790192 | Lurasidon | 488 | Industry | Multicenter (65) | N-America; Asia; Europe | Yes | Yes |
| 8 | [Kane JM](http://www.ncbi.nlm.nih.gov.proxy.library.uu.nl/pubmed/?term=Kane%20JM%5BAuthor%5D&cauthor=true&cauthor_uid=25188501), [Peters-Strickland T](http://www.ncbi.nlm.nih.gov.proxy.library.uu.nl/pubmed/?term=Peters-Strickland%20T%5BAuthor%5D&cauthor=true&cauthor_uid=25188501), [Baker RA](http://www.ncbi.nlm.nih.gov.proxy.library.uu.nl/pubmed/?term=Baker%20RA%5BAuthor%5D&cauthor=true&cauthor_uid=25188501) et al. Aripiprazole once-monthly in the acute treatment of schizophrenia: findings from a 12-week, randomized, double-blind, placebo-controlled study.  [J Clin Psychiatry.](http://www.ncbi.nlm.nih.gov.proxy.library.uu.nl/pubmed/?term=NCT01663532) 2014 Nov;75(11):1254-60. | 01663532 | Aripiprazole | 340 | Industry | Multicenter (49) | N-America; Europe | No | Yes |
| 9 | [Nasrallah HA](http://www.ncbi.nlm.nih.gov.proxy.library.uu.nl/pubmed/?term=Nasrallah%20HA%5BAuthor%5D&cauthor=true&cauthor_uid=24955752), [Cucchiaro JB](http://www.ncbi.nlm.nih.gov.proxy.library.uu.nl/pubmed/?term=Cucchiaro%20JB%5BAuthor%5D&cauthor=true&cauthor_uid=24955752), [Mao Y](http://www.ncbi.nlm.nih.gov.proxy.library.uu.nl/pubmed/?term=Mao%20Y%5BAuthor%5D&cauthor=true&cauthor_uid=24955752) et al. Lurasidone for the treatment of depressive symptoms in schizophrenia: analysis of 4 pooled, 6-week, placebo-controlled studies. [CNS Spectr.](http://www.ncbi.nlm.nih.gov.proxy.library.uu.nl/pubmed/?term=NCT00549718) 2015 Apr;20(2):140-7. | 00549718 | Lurasidone | 1330 | Industry | Multicenter (48) | N-America; Europe; Asia | Yes | Yes |
| 10 | [Stahl SM](http://www.ncbi.nlm.nih.gov.proxy.library.uu.nl/pubmed/?term=Stahl%20SM%5BAuthor%5D&cauthor=true&cauthor_uid=23541189), [Cucchiaro J](http://www.ncbi.nlm.nih.gov.proxy.library.uu.nl/pubmed/?term=Cucchiaro%20J%5BAuthor%5D&cauthor=true&cauthor_uid=23541189), [Simonelli D](http://www.ncbi.nlm.nih.gov.proxy.library.uu.nl/pubmed/?term=Simonelli%20D%5BAuthor%5D&cauthor=true&cauthor_uid=23541189) et al. Effectiveness of lurasidone for patients with schizophrenia following 6 weeks of acute treatment with lurasidone, olanzapine, or placebo: a 6-month, open-label, extension study. [J Clin Psychiatry.](http://www.ncbi.nlm.nih.gov.proxy.library.uu.nl/pubmed/?term=stahl++effectiveness+of+lurasidone+for+patients) 2013 May;74(5):507-15 | 00615433 | Lurasidone | 478 | Industry | Multicenter (52) | N-America; S-America; Asia; Europe | Yes | Yes |

| 11 | [Loebel A](http://www.ncbi.nlm.nih.gov.proxy.library.uu.nl/pubmed/?term=Loebel%20A%5BAuthor%5D&cauthor=true&cauthor_uid=23583011); [Cucchiaro J](http://www.ncbi.nlm.nih.gov.proxy.library.uu.nl/pubmed/?term=Cucchiaro%20J%5BAuthor%5D&cauthor=true&cauthor_uid=23583011), [Xu J](http://www.ncbi.nlm.nih.gov.proxy.library.uu.nl/pubmed/?term=Xu%20J%5BAuthor%5D&cauthor=true&cauthor_uid=23583011) et al. Effectiveness of lurasidone vs. quetiapine XR for relapse prevention in schizophrenia: a 12-month, double-blind, noninferiority study. [Schizophr Res.](http://www.ncbi.nlm.nih.gov.proxy.library.uu.nl/pubmed/23583011) 2013 Jun;147(1):95-102. | 00789698 | Lurasidone | 488 | Industry | Multicenter (65) | N-America; S-America; Asia; Europe | Yes | Yes |
| --- | --- | --- | --- | --- | --- | --- | --- | --- | --- |
| 12 | [Berwaerts J](http://www.ncbi.nlm.nih.gov.proxy.library.uu.nl/pubmed/?term=Berwaerts%20J%5BAuthor%5D&cauthor=true&cauthor_uid=25820612), [Liu Y](http://www.ncbi.nlm.nih.gov.proxy.library.uu.nl/pubmed/?term=Liu%20Y%5BAuthor%5D&cauthor=true&cauthor_uid=25820612), [Gopal S](http://www.ncbi.nlm.nih.gov.proxy.library.uu.nl/pubmed/?term=Gopal%20S%5BAuthor%5D&cauthor=true&cauthor_uid=25820612) et al. Efficacy and Safety of the 3-Month Formulation of Paliperidone Palmitate vs Placebo for Relapse Prevention of Schizophrenia: A Randomized Clinical Trial. [JAMA Psychiatry.](http://www.ncbi.nlm.nih.gov.proxy.library.uu.nl/pubmed/?term=berwaerts+efficacy+and+safety+3-month) 2015 Aug;72(8):830-9. | 01529515 | Paliperidone | 305 | Industry | Multicenter (56) | N-America; S-America; Asia; Europe | No | Yes |
| 13 | [Canuso CM](http://www.ncbi.nlm.nih.gov.proxy.library.uu.nl/pubmed/?term=Canuso%20CM%5BAuthor%5D&cauthor=true&cauthor_uid=20216424), [Grinspan A](http://www.ncbi.nlm.nih.gov.proxy.library.uu.nl/pubmed/?term=Grinspan%20A%5BAuthor%5D&cauthor=true&cauthor_uid=20216424), [Kalali A](http://www.ncbi.nlm.nih.gov.proxy.library.uu.nl/pubmed/?term=Kalali%20A%5BAuthor%5D&cauthor=true&cauthor_uid=20216424) et al. Medication satisfaction in schizophrenia: a blinded-initiation study of paliperidone extended release in patients suboptimally responsive to risperidone. [Int Clin Psychopharmacol.](http://www.ncbi.nlm.nih.gov.proxy.library.uu.nl/pubmed/?term=NCT00535132) 2010 May;25(3):155-64. | 00535132 | Paliperidone | 201 | Industry | Multicenter (42) | N-America; S-America; Europe | No | Yes |
| 14 | [Alphs L](http://www.ncbi.nlm.nih.gov.proxy.library.uu.nl/pubmed/?term=Alphs%20L%5BAuthor%5D&cauthor=true&cauthor_uid=25938474), [Benson C](http://www.ncbi.nlm.nih.gov.proxy.library.uu.nl/pubmed/?term=Benson%20C%5BAuthor%5D&cauthor=true&cauthor_uid=25938474), [Cheshire-Kinney K](http://www.ncbi.nlm.nih.gov.proxy.library.uu.nl/pubmed/?term=Cheshire-Kinney%20K%5BAuthor%5D&cauthor=true&cauthor_uid=25938474) et al. Real-world outcomes of paliperidone palmitate compared to daily oral antipsychotic therapy in schizophrenia: a randomized, open-label, review board-blinded 15-month study. [J Clin Psychiatry.](http://www.ncbi.nlm.nih.gov.proxy.library.uu.nl/pubmed/?term=alphs+benson+real-world) 2015 May;76(5):554-61. | 01157351 | Paliperidone | 450 | Industry | Multicenter (56) | N-America | No | Yes |
| 15 | [Macfadden W](http://www.ncbi.nlm.nih.gov.proxy.library.uu.nl/pubmed/?term=Macfadden%20W%5BAuthor%5D&cauthor=true&cauthor_uid=21191530), [Ma YW](http://www.ncbi.nlm.nih.gov.proxy.library.uu.nl/pubmed/?term=Ma%20YW%5BAuthor%5D&cauthor=true&cauthor_uid=21191530), [Thomas Haskins J](http://www.ncbi.nlm.nih.gov.proxy.library.uu.nl/pubmed/?term=Thomas%20Haskins%20J%5BAuthor%5D&cauthor=true&cauthor_uid=21191530) et al. A Prospective Study Comparing the Long-term Effectiveness of Injectable Risperidone Long-acting Therapy and Oral Aripiprazole in Patients with Schizophrenia. [Psychiatry (Edgmont).](http://www.ncbi.nlm.nih.gov.proxy.library.uu.nl/pubmed/?term=NCT00299702) 2010 Nov;7(11):23-31. | 00299702 | Risperidone | 355 | Industry | Multicenter | N-America; S-America; Asia | Yes | No |

| 16 | [Savitz AJ](http://www.ncbi.nlm.nih.gov.proxy.library.uu.nl/pubmed/?term=Savitz%20AJ%5BAuthor%5D&cauthor=true&cauthor_uid=25617253), [Lane R](http://www.ncbi.nlm.nih.gov.proxy.library.uu.nl/pubmed/?term=Lane%20R%5BAuthor%5D&cauthor=true&cauthor_uid=25617253), [Nuamah I](http://www.ncbi.nlm.nih.gov.proxy.library.uu.nl/pubmed/?term=Nuamah%20I%5BAuthor%5D&cauthor=true&cauthor_uid=25617253) et al. Efficacy and safety of paliperidone extended release in adolescents with schizophrenia: a randomized, double-blind study. [J Am Acad Child Adolesc Psychiatry.](http://www.ncbi.nlm.nih.gov.proxy.library.uu.nl/pubmed/?term=NCT01009047) 2015 Feb;54(2):126-137. | 01009047 | Paliperidone | 228 | Industry | Multicenter (44) | N-America; Asia; Europe; Africa | No | No |
| --- | --- | --- | --- | --- | --- | --- | --- | --- | --- |
| 17 | [Fleischhacker WW](http://www.ncbi.nlm.nih.gov.proxy.library.uu.nl/pubmed/?term=Fleischhacker%20WW%5BAuthor%5D&cauthor=true&cauthor_uid=24925984), [Sanchez R](http://www.ncbi.nlm.nih.gov.proxy.library.uu.nl/pubmed/?term=Sanchez%20R%5BAuthor%5D&cauthor=true&cauthor_uid=24925984), [Perry PP](http://www.ncbi.nlm.nih.gov.proxy.library.uu.nl/pubmed/?term=Perry%20PP%5BAuthor%5D&cauthor=true&cauthor_uid=24925984) et al. Aripiprazole once-monthly for treatment of schizophrenia: double-blind, randomised, non-inferiority study. [Br J Psychiatry.](http://www.ncbi.nlm.nih.gov.proxy.library.uu.nl/pubmed/24925984) 2014 Aug;205(2):135-44. | 00706654 | Aripiprazole | 662 | Industry | Multicenter (98) | N-America; Europe; S-America; Asia; Africa | No | Yes |
| 18 | [Pandina GJ](http://www.ncbi.nlm.nih.gov.proxy.library.uu.nl/pubmed/?term=Pandina%20GJ%5BAuthor%5D&cauthor=true&cauthor_uid=20473057), [Lindenmayer JP](http://www.ncbi.nlm.nih.gov.proxy.library.uu.nl/pubmed/?term=Lindenmayer%20JP%5BAuthor%5D&cauthor=true&cauthor_uid=20473057), [Lull J](http://www.ncbi.nlm.nih.gov.proxy.library.uu.nl/pubmed/?term=Lull%20J%5BAuthor%5D&cauthor=true&cauthor_uid=20473057) et al. A randomized, placebo-controlled study to assess the efficacy and safety of 3 doses of paliperidone palmitate in adults with acutely exacerbated schizophrenia. [J Clin Psychopharmacol.](http://www.ncbi.nlm.nih.gov.proxy.library.uu.nl/pubmed/?term=pandina+lindenmayer+paliperidone) 2010 Jun;30(3):235-44. | 00590577 | Paliperidone | 652 | Industry | Multicenter (66) | N-America; Asia; Europe | No | Yes |
| 19 | [Kane JM](http://www.ncbi.nlm.nih.gov.proxy.library.uu.nl/pubmed/?term=Kane%20JM%5BAuthor%5D&cauthor=true&cauthor_uid=22697189), [Sanchez R](http://www.ncbi.nlm.nih.gov.proxy.library.uu.nl/pubmed/?term=Sanchez%20R%5BAuthor%5D&cauthor=true&cauthor_uid=22697189), [Perry PP](http://www.ncbi.nlm.nih.gov.proxy.library.uu.nl/pubmed/?term=Perry%20PP%5BAuthor%5D&cauthor=true&cauthor_uid=22697189) et al. Aripiprazole intramuscular depot as maintenance treatment in patients with schizophrenia: a 52-week, Multicentercenter, randomized, double-blind, placebo-controlled study. [J Clin Psychiatry.](http://www.ncbi.nlm.nih.gov.proxy.library.uu.nl/pubmed/?term=kane+sanchez+depot) 2012 May;73(5):617-24. | 00705783 | Aripiprazole | 843 | Industry | Multicenter (98) | Africa; N-America; Asia; Europe | No | Yes |
| 20 | [Riedel M](http://www.ncbi.nlm.nih.gov.proxy.library.uu.nl/pubmed/?term=Riedel%20M%5BAuthor%5D&cauthor=true&cauthor_uid=25592805), [Schmitz M](http://www.ncbi.nlm.nih.gov.proxy.library.uu.nl/pubmed/?term=Schmitz%20M%5BAuthor%5D&cauthor=true&cauthor_uid=25592805), [Østergaard PK](http://www.ncbi.nlm.nih.gov.proxy.library.uu.nl/pubmed/?term=%C3%98stergaard%20PK%5BAuthor%5D&cauthor=true&cauthor_uid=25592805) et al. Comparison of the effects of quetiapine extended-release and quetiapine immediate-release on cognitive performance, sedation and patient satisfaction in patients with schizophrenia: a randomised, double-blind, crossover study (eXtRa). [Schizophr Res.](http://www.ncbi.nlm.nih.gov.proxy.library.uu.nl/pubmed/?term=NCT01213836) 2015 Mar;162(1-3):162-8. | 01213836 | Quetiapine | 66 | Industry | Multicenter (20) | Europe | No | Yes |

| 21 | [Kinon BJ](http://www.ncbi.nlm.nih.gov.proxy.library.uu.nl/pubmed/?term=Kinon%20BJ%5BAuthor%5D&cauthor=true&cauthor_uid=19890258), [Chen L](http://www.ncbi.nlm.nih.gov.proxy.library.uu.nl/pubmed/?term=Chen%20L%5BAuthor%5D&cauthor=true&cauthor_uid=19890258), [Ascher-Svanum H](http://www.ncbi.nlm.nih.gov.proxy.library.uu.nl/pubmed/?term=Ascher-Svanum%20H%5BAuthor%5D&cauthor=true&cauthor_uid=19890258) et al. Early response to antipsychotic drug therapy as a clinical marker of subsequent response in the treatment of schizophrenia. [Neuropsychopharmacology.](http://www.ncbi.nlm.nih.gov.proxy.library.uu.nl/pubmed/?term=kinon+chen+stauffer+early+marker) 2010 Jan;35(2):581-90. | 00337662 | Olanzapine | 628 | Industry | Multicenter (34) | N-America, S-America, Europe | No | Yes |
| --- | --- | --- | --- | --- | --- | --- | --- | --- | --- |
| 22 | [Katagiri H](http://www.ncbi.nlm.nih.gov.proxy.library.uu.nl/pubmed/?term=Katagiri%20H%5BAuthor%5D&cauthor=true&cauthor_uid=23311957), [Fujikoshi S](http://www.ncbi.nlm.nih.gov.proxy.library.uu.nl/pubmed/?term=Fujikoshi%20S%5BAuthor%5D&cauthor=true&cauthor_uid=23311957), [Suzuki T](http://www.ncbi.nlm.nih.gov.proxy.library.uu.nl/pubmed/?term=Suzuki%20T%5BAuthor%5D&cauthor=true&cauthor_uid=23311957) et al. A randomized, double-blind, placebo-controlled study of rapid-acting intramuscular olanzapine in Japanese patients for schizophrenia with acute agitation. [BMC Psychiatry.](http://www.ncbi.nlm.nih.gov.proxy.library.uu.nl/pubmed/?term=NCT00970281) 2013 Jan 11;13:20 | 00970281 | Olanzapine | 91 | Industry | Multicenter (11) | Asia | No | No |
| 23 | [Di Fiorino M](http://www.ncbi.nlm.nih.gov.proxy.library.uu.nl/pubmed/?term=Di%20Fiorino%20M%5BAuthor%5D&cauthor=true&cauthor_uid=24681810), [Montagnani G](http://www.ncbi.nlm.nih.gov.proxy.library.uu.nl/pubmed/?term=Montagnani%20G%5BAuthor%5D&cauthor=true&cauthor_uid=24681810), [Trespi G](http://www.ncbi.nlm.nih.gov.proxy.library.uu.nl/pubmed/?term=Trespi%20G%5BAuthor%5D&cauthor=true&cauthor_uid=24681810) et al. Extended-release quetiapine fumarate (quetiapine XR) versus risperidone in the treatment of depressive symptoms in patients with schizoaffective disorder or schizophrenia: a randomized, open-label, parallel-group, flexible-dose study. [Int Clin Psychopharmacol.](http://www.ncbi.nlm.nih.gov.proxy.library.uu.nl/pubmed/24681810) 2014 May;29(3):166-76. | 00640562 | Quetiapine | 216 | Industry | Multicenter (21) | Europe | No | Yes |
| 24 | [Canuso CM](http://www.ncbi.nlm.nih.gov.proxy.library.uu.nl/pubmed/?term=Canuso%20CM%5BAuthor%5D&cauthor=true&cauthor_uid=20492853), [Lindenmayer JP](http://www.ncbi.nlm.nih.gov.proxy.library.uu.nl/pubmed/?term=Lindenmayer%20JP%5BAuthor%5D&cauthor=true&cauthor_uid=20492853), [Kosik-Gonzalez C](http://www.ncbi.nlm.nih.gov.proxy.library.uu.nl/pubmed/?term=Kosik-Gonzalez%20C%5BAuthor%5D&cauthor=true&cauthor_uid=20492853) et al. A randomized, double-blind, placebo-controlled study of 2 dose ranges of paliperidone extended-release in the treatment of subjects with schizoaffective disorder. [J Clin Psychiatry.](http://www.ncbi.nlm.nih.gov.proxy.library.uu.nl/pubmed/?term=NCT00397033) 2010 May;71(5):587-98. | 00397033 | Paliperidone | 316 | Industry | Multicenter (44) | Europe; Asia; N-America | No | Yes |

| 25 | [Naber D](http://www.ncbi.nlm.nih.gov.proxy.library.uu.nl/pubmed/?term=Naber%20D%5BAuthor%5D&cauthor=true&cauthor_uid=23953270), [Peuskens J](http://www.ncbi.nlm.nih.gov.proxy.library.uu.nl/pubmed/?term=Peuskens%20J%5BAuthor%5D&cauthor=true&cauthor_uid=23953270), [Schwarzmann N](http://www.ncbi.nlm.nih.gov.proxy.library.uu.nl/pubmed/?term=Schwarzmann%20N%5BAuthor%5D&cauthor=true&cauthor_uid=23953270) et al. Subjective well-being in schizophrenia: a randomised controlled open-label 12-month non-inferiority study comparing quetiapine XR with risperidone (RECOVER). [Eur Neuropsychopharmacol.](http://www.ncbi.nlm.nih.gov.proxy.library.uu.nl/pubmed/?term=NCT00600756) 2013 Oct;23(10):1257-69. | 00600756 | Quetiapine | 798 | Industry | Multicenter (125) | Europe; S-America | No | Yes |
| --- | --- | --- | --- | --- | --- | --- | --- | --- | --- |
| 26 | [Thomas SH](http://www.ncbi.nlm.nih.gov.proxy.library.uu.nl/pubmed/?term=Thomas%20SH%5BAuthor%5D&cauthor=true&cauthor_uid=20384598), [Drici MD](http://www.ncbi.nlm.nih.gov.proxy.library.uu.nl/pubmed/?term=Drici%20MD%5BAuthor%5D&cauthor=true&cauthor_uid=20384598), [Hall GC](http://www.ncbi.nlm.nih.gov.proxy.library.uu.nl/pubmed/?term=Hall%20GC%5BAuthor%5D&cauthor=true&cauthor_uid=20384598) et al. Safety of sertindole versus risperidone in schizophrenia: principal results of the sertindole cohort prospective study (SCoP). [Acta Psychiatr Scand.](http://www.ncbi.nlm.nih.gov.proxy.library.uu.nl/pubmed/20384598) 2010 Nov;122(5):345-55. | 00856583 | Sertindole | 9858 | Industry | Multicenter | Europa; Asia | No | No |
| 27 | [Fu DJ](http://www.ncbi.nlm.nih.gov.proxy.library.uu.nl/pubmed/?term=Fu%20DJ%5BAuthor%5D&cauthor=true&cauthor_uid=25562685), [Turkoz I](http://www.ncbi.nlm.nih.gov.proxy.library.uu.nl/pubmed/?term=Turkoz%20I%5BAuthor%5D&cauthor=true&cauthor_uid=25562685), [Simonson RB](http://www.ncbi.nlm.nih.gov.proxy.library.uu.nl/pubmed/?term=Simonson%20RB%5BAuthor%5D&cauthor=true&cauthor_uid=25562685) et al. Paliperidone palmitate once-monthly reduces risk of relapse of psychotic, depressive, and manic symptoms and maintains fuioning in a double-blind, randomized study of schizoaffective disorder. [J Clin Psychiatry.](http://www.ncbi.nlm.nih.gov.proxy.library.uu.nl/pubmed/?term=NCT01193153) 2015 Mar;76(3):253-62. | 01193153 | Paliperidone | 667 | Industry | Multicenter (84) | N-America; Asia; Europe; Africa | Yes | Yes |
| 28 | [Lindenmayer JP](http://www.ncbi.nlm.nih.gov.proxy.library.uu.nl/pubmed/?term=Lindenmayer%20JP%5BAuthor%5D&cauthor=true&cauthor_uid=21346616), [Citrome L](http://www.ncbi.nlm.nih.gov.proxy.library.uu.nl/pubmed/?term=Citrome%20L%5BAuthor%5D&cauthor=true&cauthor_uid=21346616), [Khan A](http://www.ncbi.nlm.nih.gov.proxy.library.uu.nl/pubmed/?term=Khan%20A%5BAuthor%5D&cauthor=true&cauthor_uid=21346616) et al. A randomized, double-blind, parallel-group, fixed-dose, clinical trial of quetiapine at 600 versus 1200 mg/d for patients with treatment-resistant schizophrenia or schizoaffective disorder. [J Clin Psychopharmacol.](http://www.ncbi.nlm.nih.gov.proxy.library.uu.nl/pubmed/?term=NCT00297947) 2011 Apr;31(2):160-8. | 00297947 | Quetiapine | 60 | Other | Multicenter (2) | N-America | No | Yes |
| 29 | [Simpson GM](http://www.ncbi.nlm.nih.gov.proxy.library.uu.nl/pubmed/?term=Simpson%20GM%5BAuthor%5D&cauthor=true&cauthor_uid=16965196), [Mahmoud RA](http://www.ncbi.nlm.nih.gov.proxy.library.uu.nl/pubmed/?term=Mahmoud%20RA%5BAuthor%5D&cauthor=true&cauthor_uid=16965196), [Lasser RA](http://www.ncbi.nlm.nih.gov.proxy.library.uu.nl/pubmed/?term=Lasser%20RA%5BAuthor%5D&cauthor=true&cauthor_uid=16965196) et al. A 1-year double-blind study of 2 doses of long-acting risperidone in stable patients with schizophrenia or schizoaffective disorder. [J Clin Psychiatry.](http://www.ncbi.nlm.nih.gov.proxy.library.uu.nl/pubmed/?term=NCT00297388) 2006 Aug;67(8):1194-203. | 00297388 | Risperidone | 324 | Industry | Multicenter | Not provided | No | Yes |

| 30 | [Chan HY](http://www.ncbi.nlm.nih.gov.proxy.library.uu.nl/pubmed/?term=Chan%20HY%5BAuthor%5D&cauthor=true&cauthor_uid=17284127), [Lin WW](http://www.ncbi.nlm.nih.gov.proxy.library.uu.nl/pubmed/?term=Lin%20WW%5BAuthor%5D&cauthor=true&cauthor_uid=17284127), [Lin SK](http://www.ncbi.nlm.nih.gov.proxy.library.uu.nl/pubmed/?term=Lin%20SK%5BAuthor%5D&cauthor=true&cauthor_uid=17284127) et al. Efficacy and safety of aripiprazole in the acute treatment of schizophrenia in Chinese patients with risperidone as an active control: a randomized trial. [J Clin Psychiatry.](http://www.ncbi.nlm.nih.gov.proxy.library.uu.nl/pubmed/?term=NCT00283179) 2007 Jan;68(1):29-36. | 00283179 | Aripiprazole | 83 | Industry | Monocenter | Asia | No | No |
| --- | --- | --- | --- | --- | --- | --- | --- | --- | --- |
| 31 | [Kane JM](http://www.ncbi.nlm.nih.gov.proxy.library.uu.nl/pubmed/?term=Kane%20JM%5BAuthor%5D&cauthor=true&cauthor_uid=19906340), [Correll CU](http://www.ncbi.nlm.nih.gov.proxy.library.uu.nl/pubmed/?term=Correll%20CU%5BAuthor%5D&cauthor=true&cauthor_uid=19906340), [Goff DC](http://www.ncbi.nlm.nih.gov.proxy.library.uu.nl/pubmed/?term=Goff%20DC%5BAuthor%5D&cauthor=true&cauthor_uid=19906340) et al. A Multicentercenter, randomized, double-blind, placebo-controlled, 16-week study of adjuive aripiprazole for schizophrenia or schizoaffective disorder inadequately treated with quetiapine or risperidone monotherapy. [J Clin Psychiatry.](http://www.ncbi.nlm.nih.gov.proxy.library.uu.nl/pubmed/?term=NCT00325689) 2009 Oct;70(10):1348-57. | 00325689 | Aripiprazole | 323 | Industry | Multicenter (48) | N-America | No | Yes |
| 32 | [Subotnik KL](http://www.ncbi.nlm.nih.gov.proxy.library.uu.nl/pubmed/?term=Subotnik%20KL%5BAuthor%5D&cauthor=true&cauthor_uid=26107752), [Casaus LR](http://www.ncbi.nlm.nih.gov.proxy.library.uu.nl/pubmed/?term=Casaus%20LR%5BAuthor%5D&cauthor=true&cauthor_uid=26107752), [Ventura J](http://www.ncbi.nlm.nih.gov.proxy.library.uu.nl/pubmed/?term=Ventura%20J%5BAuthor%5D&cauthor=true&cauthor_uid=26107752) et al. Long-Acting Injectable Risperidone for Relapse Prevention and Control of Breakthrough Symptoms After a Recent First Episode of Schizophrenia. A Randomized Clinical Trial. [JAMA Psychiatry.](http://www.ncbi.nlm.nih.gov.proxy.library.uu.nl/pubmed/?term=NCT00333177) 2015 Aug;72(8):822-9. | 00333177 | Risperidone | 86 | Other; NIH; Industry | Monocenter | N-America | Yes | Yes |
| 33 | [Wang CY](http://www.ncbi.nlm.nih.gov.proxy.library.uu.nl/pubmed/?term=Wang%20CY%5BAuthor%5D&cauthor=true&cauthor_uid=20231321), [Xiang YT](http://www.ncbi.nlm.nih.gov.proxy.library.uu.nl/pubmed/?term=Xiang%20YT%5BAuthor%5D&cauthor=true&cauthor_uid=20231321), [Cai ZJ](http://www.ncbi.nlm.nih.gov.proxy.library.uu.nl/pubmed/?term=Cai%20ZJ%5BAuthor%5D&cauthor=true&cauthor_uid=20231321) et al. Risperidone maintenance treatment in schizophrenia: a randomized, controlled trial. [Am J Psychiatry.](http://www.ncbi.nlm.nih.gov.proxy.library.uu.nl/pubmed/?term=NCT00848432) 2010 Jun;167(6):676-85. | 00848432 | Risperidone | 404 | Other | Monocenter | Asia | No | Yes |
| 34 | [Ganguli R](http://www.ncbi.nlm.nih.gov.proxy.library.uu.nl/pubmed/?term=Ganguli%20R%5BAuthor%5D&cauthor=true&cauthor_uid=18590519), [Brar JS](http://www.ncbi.nlm.nih.gov.proxy.library.uu.nl/pubmed/?term=Brar%20JS%5BAuthor%5D&cauthor=true&cauthor_uid=18590519), [Mahmoud R](http://www.ncbi.nlm.nih.gov.proxy.library.uu.nl/pubmed/?term=Mahmoud%20R%5BAuthor%5D&cauthor=true&cauthor_uid=18590519) et al. Assessment of strategies for switching patients from olanzapine to risperidone: a randomized, open-label, rater-blinded study. [BMC Med.](http://www.ncbi.nlm.nih.gov.proxy.library.uu.nl/pubmed/?term=NCT00378183) 2008 Jun 30;6:17. | 00378183 | Risperidone | 123 | Industry | Multicenter (19) | N-America | No | Yes |
| 35 | [Chang JS](http://www.ncbi.nlm.nih.gov.proxy.library.uu.nl/pubmed/?term=Chang%20JS%5BAuthor%5D&cauthor=true&cauthor_uid=18370574), [Ahn YM](http://www.ncbi.nlm.nih.gov.proxy.library.uu.nl/pubmed/?term=Ahn%20YM%5BAuthor%5D&cauthor=true&cauthor_uid=18370574), [Park HJ](http://www.ncbi.nlm.nih.gov.proxy.library.uu.nl/pubmed/?term=Park%20HJ%5BAuthor%5D&cauthor=true&cauthor_uid=18370574) et al. Aripiprazole augmentation in clozapine-treated patients with refractory schizophrenia: an 8-week, randomized, double-blind, placebo-controlled trial. [J Clin Psychiatry.](http://www.ncbi.nlm.nih.gov.proxy.library.uu.nl/pubmed/?term=NCT00328367) 2008 May;69(5):720-31. | 00328367 | Aripiprazole | 62 | Other; Industry | Monocenter | Asia | Yes | Yes |
| 36 | [Nielsen J](http://www.ncbi.nlm.nih.gov.proxy.library.uu.nl/pubmed/?term=Nielsen%20J%5BAuthor%5D&cauthor=true&cauthor_uid=25583364), [Matz J](http://www.ncbi.nlm.nih.gov.proxy.library.uu.nl/pubmed/?term=Matz%20J%5BAuthor%5D&cauthor=true&cauthor_uid=25583364), [Mittoux A](http://www.ncbi.nlm.nih.gov.proxy.library.uu.nl/pubmed/?term=Mittoux%20A%5BAuthor%5D&cauthor=true&cauthor_uid=25583364) et al. Cardiac effects of sertindole and quetiapine: analysis of ECGs from a randomized double-blind study in patients with schizophrenia. [Eur Neuropsychopharmacol.](http://www.ncbi.nlm.nih.gov.proxy.library.uu.nl/pubmed/?term=NCT00654706) 2015 Mar;25(3):303-11. | 00654706 | Sertindole and  Quetiapine | 264 | Industry | Multicenter (26) | N-America | Yes | Yes |
| 37 | [Canuso CM](http://www.ncbi.nlm.nih.gov.proxy.library.uu.nl/pubmed/?term=Canuso%20CM%5BAuthor%5D&cauthor=true&cauthor_uid=19411369), [Dirks B](http://www.ncbi.nlm.nih.gov.proxy.library.uu.nl/pubmed/?term=Dirks%20B%5BAuthor%5D&cauthor=true&cauthor_uid=19411369), [Carothers J](http://www.ncbi.nlm.nih.gov.proxy.library.uu.nl/pubmed/?term=Carothers%20J%5BAuthor%5D&cauthor=true&cauthor_uid=19411369) et al. Randomized, double-blind, placebo-controlled study of paliperidone extended-release and quetiapine in patients with recently exacerbated schizophrenia. [Am J Psychiatry.](http://www.ncbi.nlm.nih.gov.proxy.library.uu.nl/pubmed/?term=NCT00334126) 2009 Jun;166(6):691-701. | 00334126 | Paliperidone | 399 | Industry | Not provided | Asia; Europe; N-America | No | Yes |
| 38 | [Honer WG](http://www.ncbi.nlm.nih.gov.proxy.library.uu.nl/pubmed/?term=Honer%20WG%5BAuthor%5D&cauthor=true&cauthor_uid=16452559), [Thornton AE](http://www.ncbi.nlm.nih.gov.proxy.library.uu.nl/pubmed/?term=Thornton%20AE%5BAuthor%5D&cauthor=true&cauthor_uid=16452559), [Chen EY](http://www.ncbi.nlm.nih.gov.proxy.library.uu.nl/pubmed/?term=Chen%20EY%5BAuthor%5D&cauthor=true&cauthor_uid=16452559) et al. Clozapine alone versus clozapine and risperidone with refractory schizophrenia. [N Engl J Med.](http://www.ncbi.nlm.nih.gov.proxy.library.uu.nl/pubmed/?term=NCT00272584) 2006 Feb 2;354(5):472-82. | 00272584 | Clozapine | 68 | Other | Monocenter | N-America | No | Yes |
| 39 | Durgam S, Starace A, Li D, Migliore R et al. An evaluation of the safety and efficacy of cariprazine in patients with acute exacerbation of schizophrenia: a phase II, randomized clinical trial. Schizophr Res. 2014 Feb;152(2-3):450-7. | 00694707 | Cariprazine | 732 | Industry | Multicenter (65) | N-America, Asia, Europe | No | Yes |
| 40 | [Kim SW](http://www.ncbi.nlm.nih.gov.proxy.library.uu.nl/pubmed/?term=Kim%20SW%5BAuthor%5D&cauthor=true&cauthor_uid=22809972), [Chung YC](http://www.ncbi.nlm.nih.gov.proxy.library.uu.nl/pubmed/?term=Chung%20YC%5BAuthor%5D&cauthor=true&cauthor_uid=22809972), [Lee YH](http://www.ncbi.nlm.nih.gov.proxy.library.uu.nl/pubmed/?term=Lee%20YH%5BAuthor%5D&cauthor=true&cauthor_uid=22809972) et al. Paliperidone ER versus risperidone for neurocognitive fuion in patients with schizophrenia: a randomized, open-label, controlled trial. [Int Clin Psychopharmacol.](http://www.ncbi.nlm.nih.gov.proxy.library.uu.nl/pubmed/?term=NCT00827840) 2012 Sep;27(5):267-74. | 00827840 | Paliperidone | 58 | Other; Industry | Monocenter | Asia | No | Yes |

| 41 | [Naber D](http://www.ncbi.nlm.nih.gov.proxy.library.uu.nl/pubmed/?term=Naber%20D%5BAuthor%5D&cauthor=true&cauthor_uid=26232241), [Hansen K](http://www.ncbi.nlm.nih.gov.proxy.library.uu.nl/pubmed/?term=Hansen%20K%5BAuthor%5D&cauthor=true&cauthor_uid=26232241), [Forray C](http://www.ncbi.nlm.nih.gov.proxy.library.uu.nl/pubmed/?term=Forray%20C%5BAuthor%5D&cauthor=true&cauthor_uid=26232241) et al. Qualify: a randomized head-to-head study of aripiprazole once-monthly and paliperidone palmitate in the treatment of schizophrenia. [Schizophr Res.](http://www.ncbi.nlm.nih.gov.proxy.library.uu.nl/pubmed/?term=NCT01795547) 2015 Oct;168(1-2):498-504. | 01795547 | Aripiprazole | 295 | Industry | Multicenter (2) | N-America | No | Yes |
| --- | --- | --- | --- | --- | --- | --- | --- | --- | --- |
| 42 | [Kane JM](http://www.ncbi.nlm.nih.gov.proxy.library.uu.nl/pubmed/?term=Kane%20JM%5BAuthor%5D&cauthor=true&cauthor_uid=25682550), [Skuban A](http://www.ncbi.nlm.nih.gov.proxy.library.uu.nl/pubmed/?term=Skuban%20A%5BAuthor%5D&cauthor=true&cauthor_uid=25682550), [Ouyang J](http://www.ncbi.nlm.nih.gov.proxy.library.uu.nl/pubmed/?term=Ouyang%20J%5BAuthor%5D&cauthor=true&cauthor_uid=25682550) et al. A Multicentercenter, randomized, double-blind, controlled phase 3 trial of fixed-dose brexpiprazole for the treatment of adults with acute schizophrenia. [Schizophr Res.](http://www.ncbi.nlm.nih.gov.proxy.library.uu.nl/pubmed/?term=NCT01393613) 2015 May;164(1-3):127-35. | 01393613 | Brexpiprazole | 674 | Industry | Multicenter (70) | N-America, S-America, Europe, Asia | No | Yes |
| 43 | [Correll CU](http://www.ncbi.nlm.nih.gov.proxy.library.uu.nl/pubmed/?term=Correll%20CU%5BAuthor%5D&cauthor=true&cauthor_uid=25882325), [Skuban A](http://www.ncbi.nlm.nih.gov.proxy.library.uu.nl/pubmed/?term=Skuban%20A%5BAuthor%5D&cauthor=true&cauthor_uid=25882325), [Ouyang J](http://www.ncbi.nlm.nih.gov.proxy.library.uu.nl/pubmed/?term=Ouyang%20J%5BAuthor%5D&cauthor=true&cauthor_uid=25882325) et al. Efficacy and Safety of Brexpiprazole for the Treatment of Acute Schizophrenia: A 6-Week Randomized, Double-Blind, Placebo-Controlled Trial. [Am J Psychiatry.](http://www.ncbi.nlm.nih.gov.proxy.library.uu.nl/pubmed/?term=NCT01396421) 2015 Sep 1;172(9):870-80. | 01396421 | Brexpiprazole | 636 | Industry | Multicenter (6) | N-America, Europe, Asia | No | Yes |
| 44 | [Honer WG](http://www.ncbi.nlm.nih.gov.proxy.library.uu.nl/pubmed/?term=Honer%20WG%5BAuthor%5D&cauthor=true&cauthor_uid=21733490), [MacEwan GW](http://www.ncbi.nlm.nih.gov.proxy.library.uu.nl/pubmed/?term=MacEwan%20GW%5BAuthor%5D&cauthor=true&cauthor_uid=21733490), [Gendron A](http://www.ncbi.nlm.nih.gov.proxy.library.uu.nl/pubmed/?term=Gendron%20A%5BAuthor%5D&cauthor=true&cauthor_uid=21733490) et al. A randomized, double-blind, placebo-controlled study of the safety and tolerability of high-dose quetiapine in patients with persistent symptoms of schizophrenia or schizoaffective disorder. [J Clin Psychiatry.](http://www.ncbi.nlm.nih.gov.proxy.library.uu.nl/pubmed/?term=NCT00328978) 2012 Jan;73(1):13-20. | 00328978 | Quetiapine | 131 | Industry | Multicenter (17) | N-America | No | No |
| 45 | [Chen EY](http://www.ncbi.nlm.nih.gov.proxy.library.uu.nl/pubmed/?term=Chen%20EY%5BAuthor%5D&cauthor=true&cauthor_uid=20724402), [Hui CL](http://www.ncbi.nlm.nih.gov.proxy.library.uu.nl/pubmed/?term=Hui%20CL%5BAuthor%5D&cauthor=true&cauthor_uid=20724402), [Lam MM](http://www.ncbi.nlm.nih.gov.proxy.library.uu.nl/pubmed/?term=Lam%20MM%5BAuthor%5D&cauthor=true&cauthor_uid=20724402) et al. Maintenance treatment with quetiapine versus discontinuation after one year of treatment in patients with remitted first episode psychosis: randomised controlled trial. [BMJ.](http://www.ncbi.nlm.nih.gov.proxy.library.uu.nl/pubmed/?term=NCT00334035) 2010 Aug 19;341:c4024. | 00334035 | Quetiapine | 178 | Other | Monocenter | Asia | No | Yes |

| 46 | [Fleischhacker WW](http://www.ncbi.nlm.nih.gov.proxy.library.uu.nl/pubmed/?term=Fleischhacker%20WW%5BAuthor%5D&cauthor=true&cauthor_uid=20459883), [Heikkinen ME](http://www.ncbi.nlm.nih.gov.proxy.library.uu.nl/pubmed/?term=Heikkinen%20ME%5BAuthor%5D&cauthor=true&cauthor_uid=20459883), [Olié JP](http://www.ncbi.nlm.nih.gov.proxy.library.uu.nl/pubmed/?term=Oli%C3%A9%20JP%5BAuthor%5D&cauthor=true&cauthor_uid=20459883) et al. Effects of adjuive treatment with aripiprazole on body weight and clinical efficacy in schizophrenia patients treated with clozapine: a randomized, double-blind, placebo-controlled trial. [Int J Neuropsychopharmacol.](http://www.ncbi.nlm.nih.gov.proxy.library.uu.nl/pubmed/?term=NCT00300846) 2010 Sep;13(8):1115-25. | 00300846 | Aripiprazole | 207 | Industry | Multicenter (57) | Europe, Africa | Yes | No |
| --- | --- | --- | --- | --- | --- | --- | --- | --- | --- |
| 47 | Strom BL, Eng SM, Faich G et al. Comparative mortality associated with ziprasidone and olanzapine in real-world use among 18,154 patients with schizophrenia: The Ziprasidone Observational Study of Cardiac Outcomes (ZODIAC). Am J Psychiatry. 2011 Feb;168(2):193-201. | 00418171 | Ziprasidone | 18,154 | Industry | Multicenter (5) | N-America | No | No |
| 48 | Berger GE, Proffitt TM, McConchie M et al. Dosing quetiapine in drug-naive first-episode psychosis: a controlled, double-blind, randomized, single-center study investigating efficacy, tolerability, and safety of 200 mg/day vs. 400 mg/day of quetiapine fumarate in 141 patients aged 15 to 25 years. J Clin Psychiatry. 2008 Nov;69(11):1702-14. | 00449397 | Quetiapine | 141 | Industry | Monocenter | Australia | Yes | Yes |
