# Supplementary material for: Outcome reporting bias in randomized-controlled trials investigating antipsychotic drugs
Source: Transl Psychiatry. 2017 Sep 12;7(9):e1232–. doi: 10.1038/tp.2017.203 (PMC5639247; doi:10.1038/tp.2017.203)
Supplement: Supplementary Table 3 [file tp2017203x3.docx]

| **Study No.** | **Study Reference** | **NCT Number** | **Type of antipsychotic** | **Sample size in publication** | **Funding** | **Mono-/ multicenter** | **Continent(s)** | **Discrepancies in primary outcome(s)** | **Discrepancies in secondary outcome(s)** |
| --- | --- | --- | --- | --- | --- | --- | --- | --- | --- |
| 1 | Alphs L, Fu DJ, Turkoz I. Paliperidone for the treatment of schizoaffective disorder. Expert Opin Pharmacother 2016; 17(6): 871-83. | 00412373 | Paliperidone | 311 | Industry | Multicenter (44) | N-America; Europe; Asia | Yes | Yes |
| 2 | Durgam S, Cutler AJ, Lu K, et al. Cariprazine in acute exacerbation of schizophrenia: a fixed-dose, phase 3, randomized, double-blind, placebo- and active-controlled trial. J Clin Psychiatry 2015; 76(12): e1574-82. | 01104766 | Cariprazine | 617 | Industry | Multicenter (57) | N-America; Europe | No | Yes |
| 3 | Durgam S, Earley W, Li R, et al. Long-term cariprazine treatment for the prevention of relapse in patients with schizophrenia: A randomized, double-blind, placebo-controlled trial. Schizophr Res 2016; 176(2-3): 264-71. | 01412060 | Cariprazine | 765 | Industry | Multicenter (72) | N-America; Europe; Asia | No | Yes |
| 4 | Grunder G, Heinze M, Cordes J, et al. Effects of first-generation antipsychotics versus second-generation antipsychotics on quality of life in schizophrenia: a double-blind, randomised study. Lancet Psychiatry 2016; 3(8): 717-29. | 01164059 | Olanzapine, Flupentixol, Quetiapine, Aripiprazole, Haloperidol | 149 | Other | Multicenter (14) | Europe | No | Yes |
| 5 | Weiden PJ, Manning R, Wolfgang CD, et al. A Randomized Trial of Iloperidone for Prevention of Relapse in Schizophrenia: The REPRIEVE Study. CNS Drugs 2016; 30(8): 735-47. | 01291511 | Iloperidone | 303 | Industry | Multicenter (66) | N-America; Europe; Asia | No | Yes |
| 6 | Loebel A, Silva R, Goldman R, et al. Lurasidone Dose Escalation in Early Nonresponding Patients With Schizophrenia: A Randomized, Placebo-Controlled Study. J Clin Psychiatry 2016; 77(12): 1672-80. | 01821378 | Lurisadone | 412 | Industry | Multicenter (66) | N-America; S-America; Europe | No | Yes |
| 7 | Robinson DG, Gallego JA, John M, et al. A Randomized Comparison of Aripiprazole and Risperidone for the Acute Treatment of First-Episode Schizophrenia and Related Disorders: 3-Month Outcomes. Schizophr Bull 2015; 41(6): 1227-36. | 00320671 | Aripiprazole, Risperidone | 198 | Other | Multicenter (8) | N-America | No | Yes |
