# Supplementary material for: Outcome reporting bias in randomized-controlled trials investigating antipsychotic drugs
Source: Transl Psychiatry. 2017 Sep 12;7(9):e1232–. doi: 10.1038/tp.2017.203 (PMC5639247; doi:10.1038/tp.2017.203)
Supplement: Supplementary Table 4 [file tp2017203x4.docx]

**Supplementary Table 4: Discrepancies between prespecified and published primary/secondary outcomes**

|  | No. of trials with discrepancies (%) | Total no. of discrepancies (% of prespecified/published outcomes) | Mean no. of discrepancies per trial (N=7) |
| --- | --- | --- | --- |
| Primary outcomes | | | |
| Prespecified outcome not published | 0 (0%) | 0/9 (0%) | 0 |
| Prespecified outcome converted to secondary outcome in publication | 1 (14%) | 1/9 (11%) | 0.14 |
| Published outcomes not prespecified | 0 (0%) | 0/9 (0%) | 0 |
| Secondary outcomes | | | |
| Prespecified outcome not published | 4 (57%) | 18/35 (51%) | 2.6 |
| Prespecified outcome converted to primary outcome in publication | 0 (0%) | 0/35 (0%) | 0 |
| Published outcomes not prespecified | 7 (100%) | 19/38 (50%) | 2.7 |
